# RETRACTED: Chuang et al. Neutrophil–Lymphocyte Ratio as a Predictor of Cerebral Small Vessel Disease in a Geriatric Community: The I-Lan Longitudinal Aging Study. *Brain Sci.* 2023, *13*, 1087

**DOI:** 10.3390/brainsci15111246

**Published:** 2025-11-20

**Authors:** Shao-Yuan Chuang, Yin-Chen Hsu, Kuang-Wei Chou, Kuo-Song Chang, Chiong-Hee Wong, Ya-Hui Hsu, Hao-Min Cheng, Chien-Wei Chen, Pang-Yen Chen

**Affiliations:** 1 Institute of Population Health Science, National Health Research Institute, Miaoli 36001, Taiwan; chuangsy@nhri.edu.tw; 2 Institute of Public Health, National Yang Ming Chiao Tung University School of Medicine, Taipei 30010, Taiwan; hmcheng@vghtpe.gov.tw; 3 Department of Nursing, Yuanpei University of Medical Technology, Hsinchu 30015, Taiwan; ymsc10014@gmail.com (Y.-C.H.); arayboys@gmail.com (K.-W.C.); a0975630558@gmail.com (Y.-H.H.); 4 Department of Diagnostic Radiology, Chang Gung Memorial Hospital Chiayi Branch, Chiayi 61363, Taiwan; 5 College of Medicine, Chang Gung University, Taoyuan 33302, Taiwan; 6 Department of Emergency Medicine, Mackay Memorial Hospital, Taipei 11008, Taiwan; kuosong@gmail.com (K.-S.C.); aaronwch@gmail.com (C.-H.W.); 7 Mackay Junior College of Medicine, Nursing, and Management, Taipei 11260, Taiwan; 8 Department of Internal Medicine, National Yang Ming Chiao Tung University School of Medicine, Taipei 31254, Taiwan; 9 Center for Evidence-Based Medicine, Taipei Veterans General Hospital, Taipei 11217, Taiwan; 10 Department of Medical Education, Taipei Veterans General Hospital, Taipei 11217, Taiwan

The journal retracts the article titled “Neutrophil–Lymphocyte Ratio as a Predictor of Cerebral Small Vessel Disease in a Geriatric Community: The I-Lan Longitudinal Aging Study” [1], cited above.

Following publication, concerns were raised with the Editorial Office regarding authorship issues and unauthorized use of data presented within this article [1].

Adhering to our standard procedure, the Editorial Office, Editorial Board, and National Yang Ming Chiao Tung University conducted an investigation that confirmed that a number of authors were not aware of the manuscript’s submission and subsequent publication, nor were the appropriate permission to publish the data gained. Consequently, the Editorial Board decided to retract this article as per MDPI’s retraction policy (https://www.mdpi.com/ethics#_bookmark30, accessed on 1 July 2025).

This retraction was approved by the Editor-in-Chief of the *Brain Sciences* journal.

Shao-Yuan Chuang and Hao-Min Cheng agree with this retraction. Pang-Yen Chen disagrees with this retraction. The remaining authors did not provide a comment on this decision.
